# Application of carbon nanoparticles in endoscopic thyroid cancer surgery: a systematic review and meta-analysis

**DOI:** 10.3389/fsurg.2023.1283573

**Published:** 2024-01-08

**Authors:** Jiaxi He, Pengfei Sun, Jianhuang Lin, Jiali Shen, Huihui Lin, Hongzhan Jiang, Rongliang Qiu, Ende Lin, Yizhuo Lu

**Affiliations:** ^1^ School of Medicine, Xiamen University, Xiamen, Fujian, China; ^2^ The School of Clinical Medicine, Fujian Medical University, Fuzhou, Fujian, China; ^3^ Nursing College, Fujian University of Traditional Chinese Medicine, Fuzhou, Fujian, China; ^4^ Department of General Surgery, Zhongshan Hospital, Xiamen University, Xiamen, Fujian, China

**Keywords:** carbon nanoparticles, endoscopy, meta-analysis, systematic review, thyroid cancer

## Abstract

**Background:**

There has been a substantial increase in incidence of thyroid cancer globally over the past three decades, emphasizing the necessity for efficient surgical management. Surgical intervention requires meticulous lymphatic dissection; however, it is challenging to both accurately identify lymph nodes and preserve the surrounding structures. We investigated the role of carbon nanoparticles in endoscopic thyroid cancer surgery to improve surgical effects and reduce postoperative complications.

**Methods:**

Chinese and English literature databases from inception to May 2023 were searched based on inclusion criteria, and data were extracted independently by two investigators. STATA software was used for data analysis.

**Results:**

A comprehensive systematic review and meta-analysis were conducted with 13 publications (9 randomized and 4 non-randomized controlled trials). The results demonstrated that the application of carbon nanoparticles in thyroid surgery led to an increase in the number of retrieved lymph nodes and identification of metastatic lymph nodes. Furthermore, it considerably reduced the rate of improper parathyroidectomy and the incidence of postoperative hypocalcemia.

**Conclusion:**

The application of carbon nanoparticles can effectively improve the effects of surgical treatment, can enhance the identification of intraoperative lymph nodes, reduce postoperative complications, and protect the integrity and function of the parathyroid gland.

**Systematic Review Registration:**

www.crd.york.ac.uk/PROSPERO, identifier, CRD42023420504.

## 1 Introduction

Over the past three decades, the global incidence of thyroid cancer has increased by 240% (1, 2), making it one of the most prevalent endocrine tumors (1). While most thyroid cancers are less aggressive, with a 90% chance of a 10-year survival rate (3), the risk of lymph node metastasis exists even in early-stage thyroid cancer. Between 20% and 90% of individuals undergoing therapy for thyroid carcinoma develop cervical lymph node metastases (4). Cervical lymph node metastasis is an important factor affecting the outcome of papillary thyroid cancer and is also a potential risk factor for disease recurrence (5). Therefore, thorough intraoperative lymphatic dissection plays a crucial role in the outcome and prognosis of the disease.

The continuous development of endoscopy technology has led to the widespread utilization of minimally invasive endoscopic surgery, greatly contributing to thyroid cancer management. Owing to the increasing prevalence of young patients seeking to improve cosmetic outcomes without visible scarring, endoscopic surgery has emerged as the optimal choice considering both treatment efficacy and aesthetic results. Thyroid cancer surgery entails a meticulous dissection of the affected lymph nodes while preserving the adjacent structures, such as nerves and blood vessels; thus, precise identification of lymph nodes is a prerequisite for this. Previously, the surgeon's expertise was required to accurately identify the tissues of lymph and non-lymph nodes, which made it difficult to provide consistent and reliable results. Moreover, considerable anatomical variations in the tissue surrounding the thyroid gland increase the risk of damaging the parathyroid gland and recurrent laryngeal nerve, leading to post-surgical complications such as hypocalcemia, hoarseness, and dysphagia (6). The use of carbon nanoparticle tracers in thyroid cancer endoscopic surgeries is associated with substantial improvements in accurate tumor diagnosis and resection, while reducing postoperative complications.

Carbon nanoparticles are specially treated smooth spherical particles with a diameter of 150 nm, possessing several advantages such as precise targeting, ease of use, and stability (7). They enable enhanced visualization of the thyroid tissue and its associated lymph nodes, creating a distinct contrast with the surrounding tissues, thereby tracing lymph nodes and protecting the surrounding tissue structures. Accurate identification of the lymph node and thyroid structure in the drainage area and increased lymph node clearance reduce postoperative complications caused by parathyroid miscut and laryngeal nerve injury. Thus, after being approved, carbon nanoparticle tracers have been widely employed in clinical thyroid cancer surgery.

Here, we aimed to systematically analyze and evaluate the effects of carbon nanoparticles in endoscopic thyroid cancer surgery and provide more conclusive evidence for the clinical application of carbon nanoparticle tracers.

## 2 Methods

The protocol of this systematic review (register number: CRD42023420504) was registered at PROSPERO (www.crd.york.ac.uk/PROSPERO).

### 2.1 Search strategy

Investigators searched PubMed, ClinicalTrials.gov, EMBASE, the Cochrane Database of Systematic Reviews, the China Biology Medicine Database, CNKI, the WANFANG database, and CqVIP, and compared all available literature on the use of carbon nanoparticles in endoscopic thyroid cancer surgery. The keywords “carbon nanoparticles, thyroid cancer, and endoscopy” were used to search literature in English, and those that met the criteria were included in this study.

Taking PubMed as an example, the specific search strategy: “((Endoscopy) OR (Endoscopic)) AND (((((((Nano carbon) OR (CN)) OR (CNs)) OR (CNPS)) OR (carbon nanoparticles)) OR (nano-carbon)) OR (Nanocarbon)) AND ((((((((((((((((((Thyroid Neoplasms) OR (Neoplasm, Thyroid)) OR (Thyroid Neoplasm)) OR (Neoplasms, Thyroid)) OR (Neoplasms, Thyroid)) OR (Carcinoma, Thyroid)) OR (Carcinomas, Thyroid)) OR (Thyroid Carcinomas)) OR (Cancer of Thyroid)) OR (Thyroid Cancers)) OR (Thyroid Cancer)) OR (Cancer, Thyroid)) OR (Cancers, Thyroid)) OR (Cancer of the Thyroid)) OR (Thyroid Adenoma)) OR (Adenoma, Thyroid)) OR (Adenomas, Thyroid)) OR (Thyroid Adenomas)),” was employed. We also used the “snowball” approach to trace the references included in the study.

### 2.2 Inclusion criteria

The following criteria were used to determine the choice of our reviewed studies. (1) Patients: patients who were diagnosed with thyroid cancer using preoperative puncture pathology and underwent thyroid cancer surgery for the first time. (2) Intervention: the intervention for the test group was a carbon nanoparticle tracer, while that for the control group was a blank without any tracer. (3) Outcome: the observed indicators comprised the average number of lymph nodes dissected, the average number of positive lymph nodes dissected, the average number of metastatic lymph nodes dissected, parathyroid resection, recurrent laryngeal nerve injury, and postoperative hypocalcemia. (4) Study type: randomized controlled trials, non-randomized controlled trial, and retrospective studies were used. (5) The language was Chinese or English only.

### 2.3 Exclusion criteria

We excluded research or literature pertaining to the following factors from our review: (1) open thyroid cancer surgery; (2) experimental groups combined with other study variables; (3) case reports, reviews, and meta-analysis; and (4) experiments in which animals or cells were used as research objects. Low-quality literature (Jadad scale score <4) was also excluded.

### 2.4 Data extraction and quality assessment

Two researchers were independently involved in the screening process, and relevant experimental data were extracted, including time of publication, first author, journal of publication, number of experimental and control groups, patients' sex, mean age, study methods, study index data, and outcomes. The obtained data were combined and analyzed. When there was a discrepancy in the literature extracted by the two researchers, we involved a third researcher for data extraction and screening. The extracted information and data were analyzed.

### 2.5 Statistical analysis

STATA SE 18 software (https://www.stata.com/) was used for all data analyses in this review. While continuous outcome variables were compared using the weighted mean difference (WMD), dichotomous outcome variables were compared using the odds ratio (OR). We calculated the 95% confidence interval (CI) for both WMD and OR. The statistical results were processed to determine the effect model. When *P *≥ 0.1 and *I*^2^* *≤ 50%, a fixed-effects model was used for meta-analysis. When *P *< 0.1 and *I*^2^* *> 50%, sensitivity analysis was performed on the data to evaluate the effect of each group of data on the heterogeneity of the results. If there were data with large effects, they were excluded, and then the fixed-effect analysis was performed. If there was no significant shadow heterogeneity in the data, a random-effects model was used to conduct a meta-analysis. The results were considered statistically different when *P *< 0.05. Forest plots were also drawn per the statistical results. When the number of included literatures was >10, funnel plots were drawn, and the symmetry of the funnel plots was evaluated further to analyze the publication bias of the included data.

## 3 Results

### 3.1 Study selection

**Figure 1:**
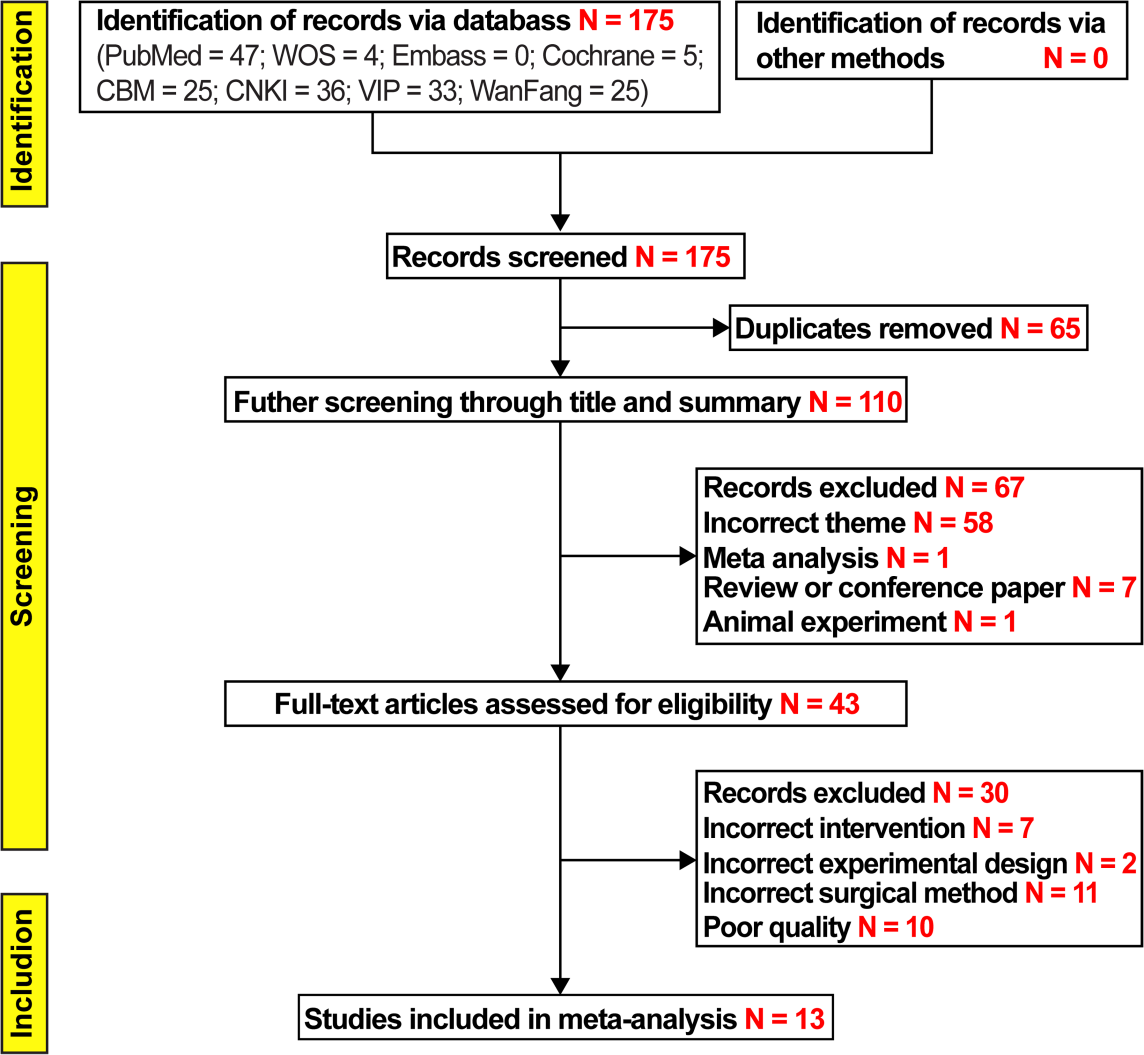
Study flow diagram.

The researchers completed literature screening per the established screening process and conditions. The flow chart of the literature screening is shown in Figure 1. Initially, 175 articles were screened based on the keywords. Based on the titles and abstract, we removed 65 duplicates, 58 non-conforming articles, 1 meta-analysis, 7 systematic reviews, and 1 animal analysis per the exclusion criteria. Based on the full-text review of the 43 articles, we further excluded 7 reports of studies with inconsistent interventions, 2 with inconsistent experimental design, 11 with inconsistent surgical methods, and 10 with poor literature quality. In total, 13 (8–20) studies were considered eligible for inclusion in this meta-analysis, comprising 4 studies reported in English and 9 studies reported in Chinese, involving a total of 1,463 patients.

### 3.2 Study characteristics

Information on the characteristics of the studies analyzed in the meta-study is included in Table 1. We used the Jadad scale (21) for nine randomized controlled trials included in this study. Four aspects of literature quality were evaluated: random sequence production (0–2), allocation concealment (0–2), blinding method (0–2), and withdrawal (0–1). While literature with a composite score of 1–3 was considered low quality, that with a score of 4–7 was considered high quality. An overview of the evaluation findings is presented in Table 2. Furthermore, we used the Newcastle–Ottawa Scale (22) for evaluating the quality of four non-randomized controlled trials, based on three evaluation aspects: selection (0–4), comparability (0–2), and exposure (0–3). Studies with a comprehensive score exceeding 5 points were considered eligible for inclusion in the meta-analysis. The evaluation results are presented in Table 3.

**Table 1:** Characteristics of the 13 studies included in the meta-analysis.

Year	Author	*N*		Sex (male/female)		Average age (years)		Study design	Concealment of allocation	Blinding	Indices
		Experimental	Control	Experimental	Experimental	Experimental	Control				
2022	Liu et al. (8)	160	134	32/128	33/101	40.64 ± 10.70	40.25 ± 10.02	NRCT	Unclear	Unblinding	①②
2020	Cheng et al. (9)	50	52	–	–	–	–	NRCT	Unclear	Unblinding	①④
2017	Chen et al. (10)	33	32	5/28	7/25	30.45 ± 6.72	32.71 ± 5.9	NRCT	Unclear	Unblinding	①②④⑥
2018	Wu et al. (11)	13	13	5/8	7/25	43.15 ± 8.97	44.08 ± 7.62	RCT	Unclear	Unclear	①④⑥
2016	Ye et al. (12)	45	40	9/36	8/32	46.89 ± 9.08	46.32 ± 9.10	RCT	Unclear	Unblinding	①③④
2013	Yang et al. (13)	21	22	1/20	1/21	32.48 ± 4.69	32.32 ± 5.35	RCT	Unclear	Unclear	①③④
2022	Cao et al. (14)	58	40	–	–	–	–	NRCT	Unclear	Unclear	①②⑤⑥
2018	You et al. (15)	60	60	18/42	20/40	35.42 ± 3.61	34.38 ± 3.52	RCT	Unclear	Unclear	①⑥
2018	Rao et al. (16)	32	40	–	–	–	–	RCT	Unclear	Unblinding	①④⑤
2020	Ma et al. (17)	51	42	8/43	5/37	31.8 ± 7.3	30.2 ± 9.2	RCT	Unclear	Unclear	①②
2021	Rao et al. (18)	50	58	9/41	16/42	46.8 ± 11.9	44.0 ± 10.2	RCT	Unclear	Unclear	①④⑤
2020	Zhang et al. (19)	152	150	9/143	13/137	33.32 ± 9.32	33.57 ± 9.62	RCT	Unclear	Unblinding	①③⑤
2015	Wang et al. (20)	28	27	1/28	2/27	30.25 ± 6.04	29.44 ± 6.27	RCT	Unclear	Unblinding	①③④

① Average number of retrieved lymph nodes; ② average number of retrieved positive lymph nodes; ③ average number of metastatic lymph nodes; ④ number of accidental parathyroid removal; ⑤ number of recurrent laryngeal nerve injuries; ⑥ number of hypocalcemia.

RCT, randomized controlled trial; NRCT, non-randomized controlled trials.

**Table 2:** Details of the quality assessment of randomized controlled trials.

Year	Author	Randomization	Concealment of allocation	Blinding	Loss to follow-up, %	Quality assessment
2018	Wu et al. (11)	No mentioned description	Only mentioned randomized	Unclear	0	4
2016	Ye et al. (12)	No mentioned description	Only mentioned randomized	Unblinding	0	3
2013	Yang et al. (13)	No mentioned description	Only mentioned randomized	Unclear	0	4
2018	You et al. (15)	No mentioned description	Only mentioned randomized	Unclear	0	4
2018	Rao et al. (16)	Random-number table	Only mentioned randomized	Unblinding	0	4
2020	Ma et al. (17)	No mentioned description	Only mentioned randomized	Unclear	0	4
2021	Rao et al. (18)	Random-number table	Only mentioned randomized	Unclear	0	5
2020	Zhang et al. (19)	Computer-generated randomization chart	Only mentioned randomized	Unblinding	0	4
2015	Wang et al. (20)	Computer-generated randomization chart	Only mentioned randomized	Unblinding	0	4

Quality assessment based on the Jadad scale.

**Table 3:** Details of the quality assessment of nonrandomized controlled trails.

Year	Author	Study design	Selection	Comparability	Exposure	Quality assessment
2022	Liu et al. (8)	Concurrent retrospective chart review	4	2	2	8
2020	Cheng et al. (9)	Concurrent retrospective chart review	4	2	2	8
2017	Chen et al. (10)	Concurrent retrospective chart review	4	2	2	8
2022	Cao et al. (14)	Prospective controlled study	4	2	2	8

Quality assessment based on the Newcastle–Ottawa Scale.

### 3.3 Average number of retrieved lymph nodes

**Figure 2:**
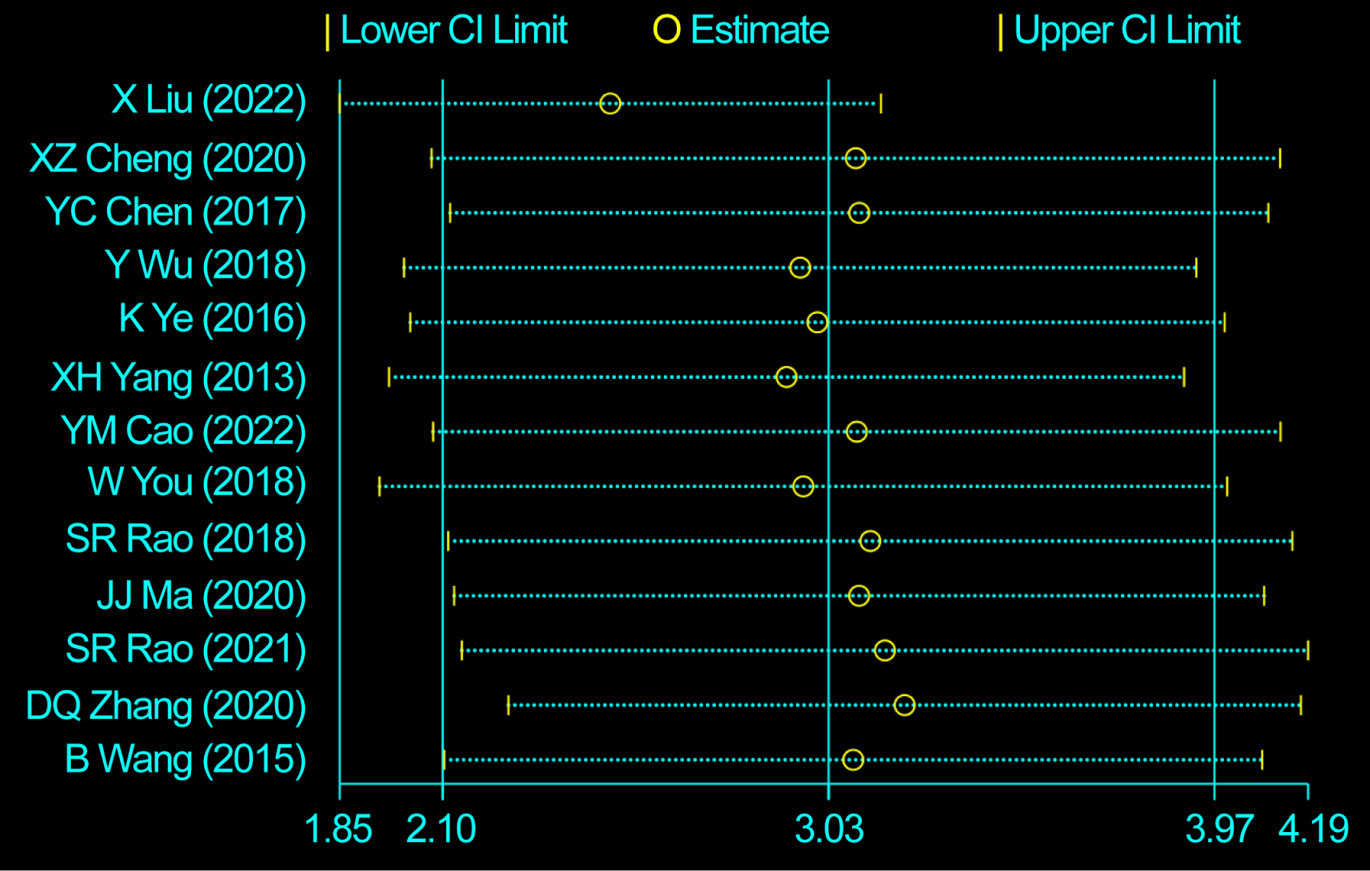
Heterogeneity test of the 13 studies included. The sensitivity analysis indicated that no study had a substantial impact on the overall research results.

**Figure 3:**
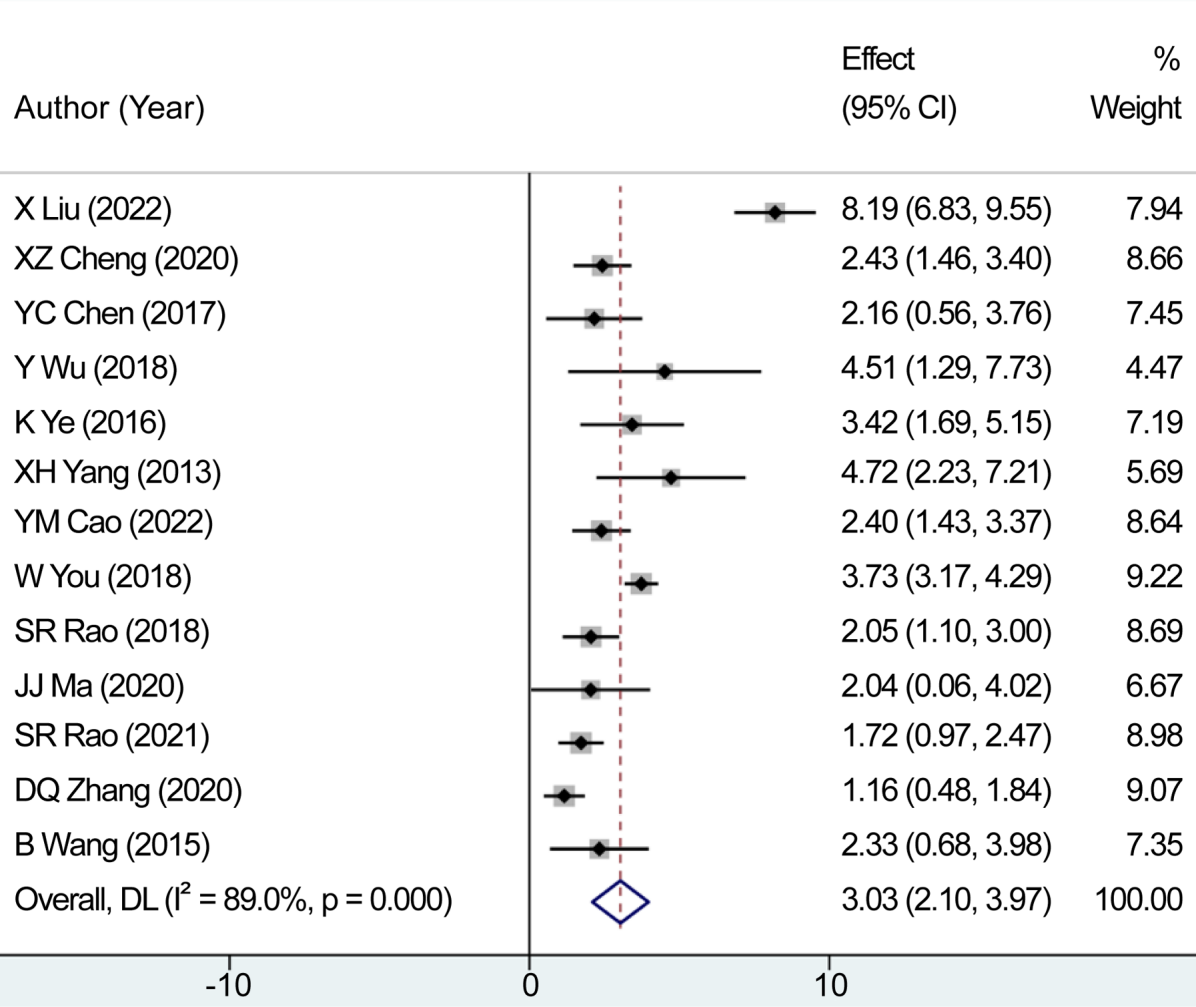
Combined effect size of the average number of retrieved lymph nodes. The carbon nanoparticle tracers had a notable effect on lymph node staining and could improve lymph node dissection efficiency.

**Figure 4:**
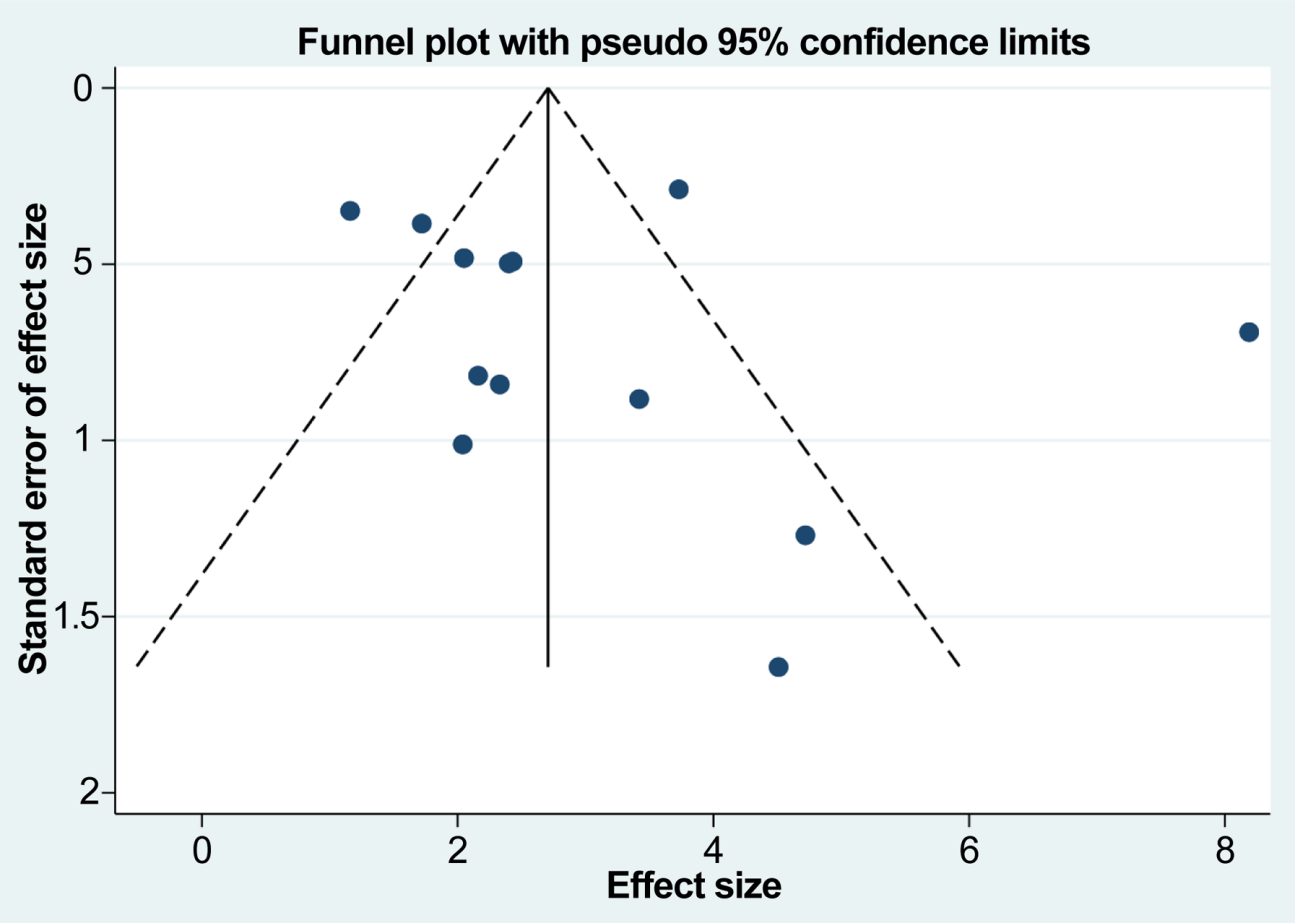
Potential publication biases among the 13 studies. The absence of publication bias in the included literature.

We evaluated 13 studies in this review to assess the number of retrieved lymph nodes. Strong heterogeneity among the studies was noted, following a heterogeneity test, where *I*^2^*^ ^*= 89.0% and *P *= 0.000 (*Q*-test). The sensitivity analysis indicated that no study had a substantial impact on the overall research results (Figure 2). For the meta-analysis, we selected random-effects modeling. The combined effect size (WMD = 3.033, 95% CI: 2.100–3.967, *P *= 0.000) demonstrated that the use of carbon nanoparticle tracers resulted in an average increase of 3.033 lymph nodes compared with that in the control group. Thus, carbon nanoparticle tracers had a notable effect on lymph node staining and could improve lymph node dissection efficiency (Figure 3). To investigate potential publication biases among the 13 literature sources, we drew a funnel diagram (Figure 4). The statistical analysis yielded a *P* value of 0.468, indicating the absence of publication bias in the included literature.

### 3.4 Average number of retrieved positive lymph nodes

**Figure 5:**
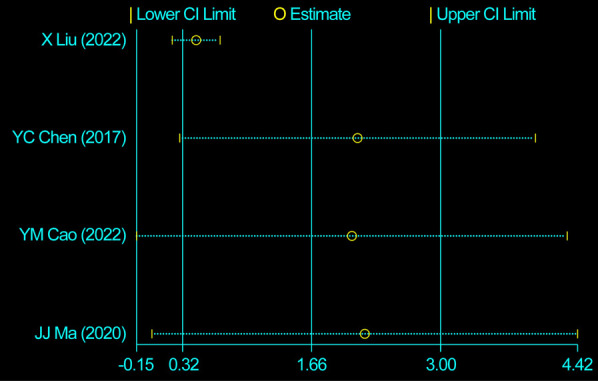
Heterogeneity test of four studies. The sensitivity analysis identified the study by Liu et al. as the source of heterogeneity.

**Figure 6:**
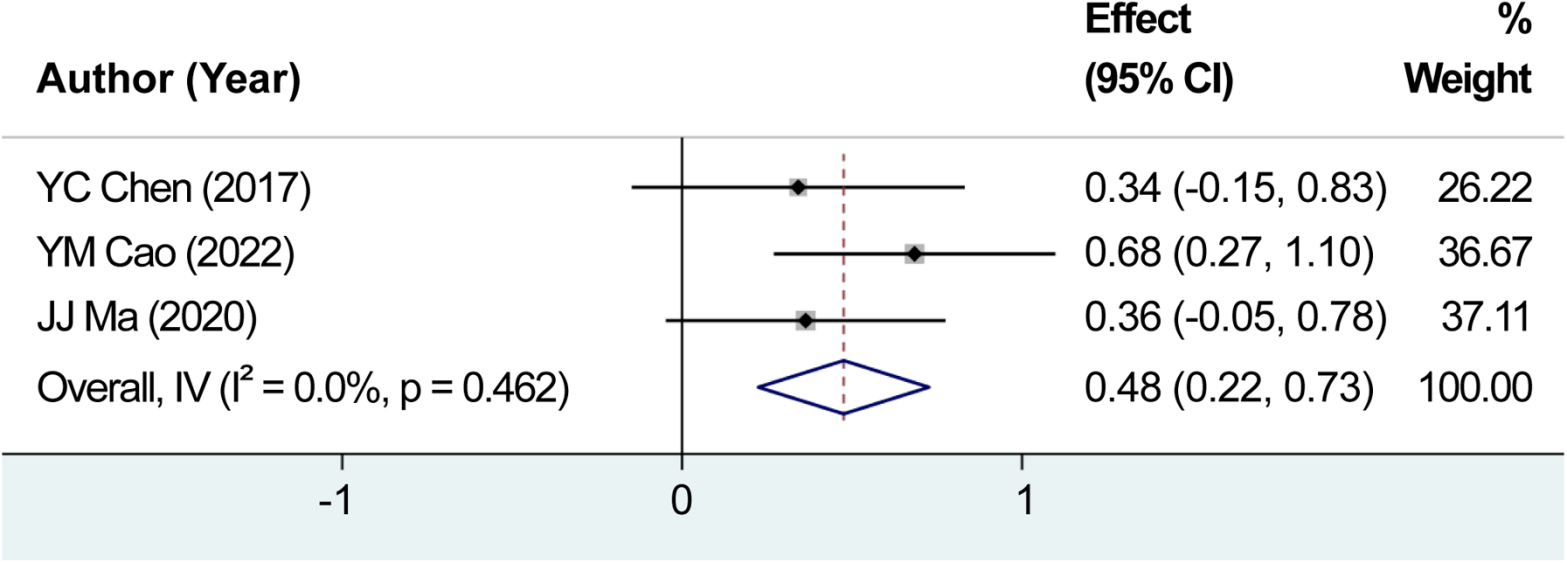
Combined effect size of the average number of retrieved positive lymph nodes. The effect size after meta-merger indicated that the application of carbon nanoparticle tracers resulted in the removal of 0.457 additional positive lymph nodes during surgery compared with that in the control group.

In this study, four studies were assessed for heterogeneity. The analysis revealed a high level of heterogeneity among the included studies with *I*^2^*^ ^*= 96.5% and a significant *Q*-test result (*P *= 0.000). The sensitivity analysis identified the study by Liu et al. (8) as the source of heterogeneity (Figure 5). Upon the exclusion of this study, no heterogeneity was observed among the remaining studies, with *I*^2^*^ ^*= 0.00% and *P *= 0.462 for Q-test. Consequently, the fixed-effects model was employed. The effect size after meta-merger (WMD = 0.457, 95% CI: 0.224–0.726, *P *= 0.000) indicated that the application of carbon nanoparticle tracers resulted in the removal of 0.457 additional positive lymph nodes during surgery compared with that in the control group. This finding suggests that the use of carbon nanoparticle tracers before surgery can assist surgeons in achieving a more thorough removal of positive lymph nodes, thereby reducing the risk of thyroid cancer recurrence to a certain extent. A visual representation of the obtained results is presented in Figure 6.

### 3.5 Average number of metastatic lymph nodes

**Figure 7:**
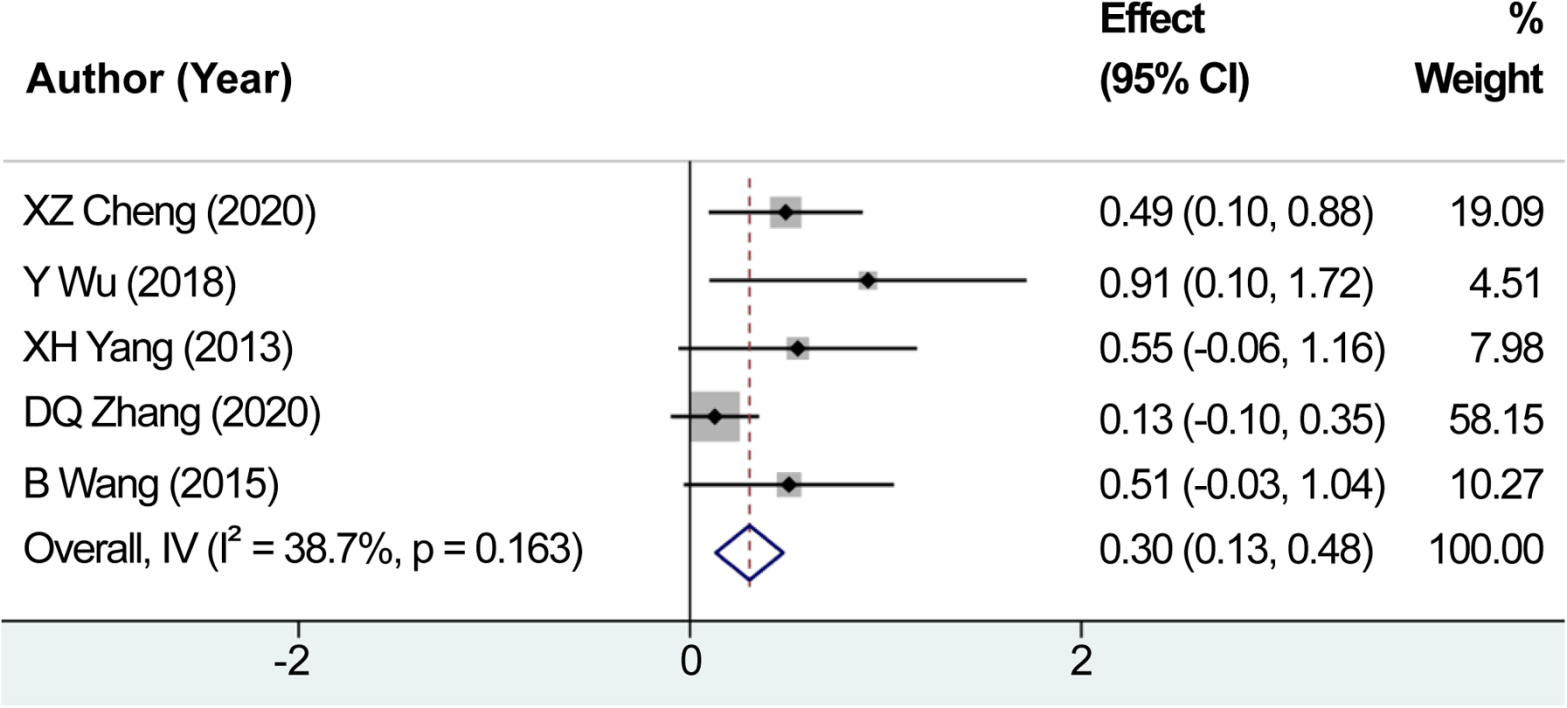
Combined effect size of the average number of metastatic lymph nodes. The combined effect size indicated that the use of carbon nanoparticle tracers led to the identification of approximately 0.304 additional metastatic lymph nodes compared to conventional methods alone.

A comprehensive review yielded a total of five relevant studies that were subsequently incorporated into this analysis. After testing for heterogeneity, *I*^2 ^= 38.7% and *P *= 0.163 for Q-test, we did not record any significant heterogeneity among the studies analyzed for the average number of metastatic lymph nodes, and the fixed-effects were selected for the combined effect size. The combined effect size (WMD = 0.304, 95% CI: 0.132–0.477, *P *= 0.001) indicated that the use of carbon nanoparticle tracers led to the identification of approximately 0.304 additional metastatic lymph nodes compared to conventional methods alone (Figure 7). These findings suggest that the implementation of carbon nanoparticles as a diagnostic tool may enhance the detection accuracy, ultimately improving overall surgical outcomes in treating thyroid cancer.

### 3.6 Number of accidental parathyroid removals

**Figure 8:**
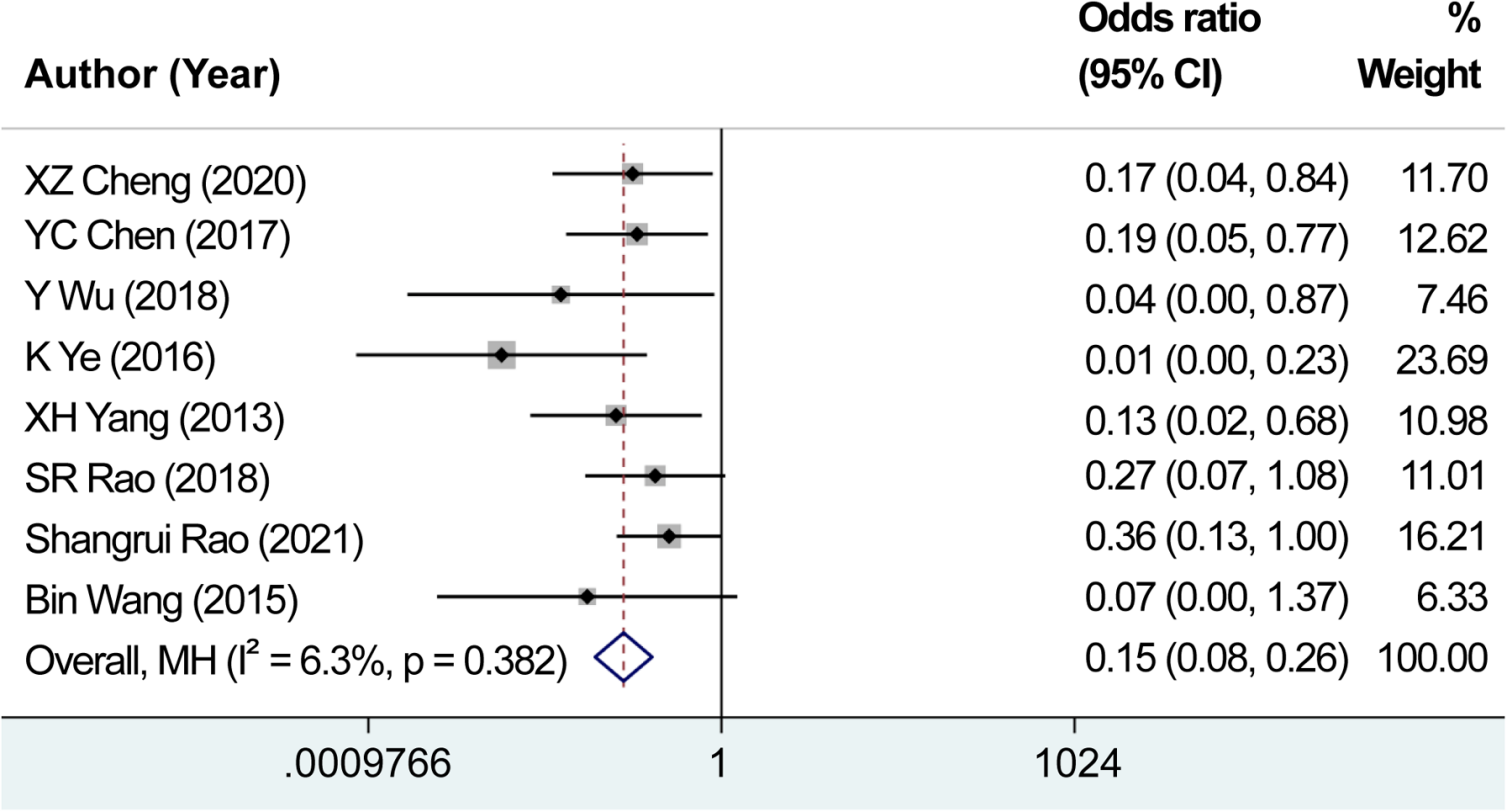
Combined effect size of the number of accidental parathyroid removals. The preoperative application of carbon nanoparticles resulted in a 14.6% reduction in the rate of parathyroid miscutting compared with that in the control group.

We evaluated eight studies focusing on the number of accidental parathyroid removals. After testing for heterogeneity, no significant heterogeneity was found among the studies, indicated by the *I*^2^ value of 6.3% and non-significant Q-test result (*P *= 0.382). Consequently, the fixed-effects model was employed for estimating the pooled effect sizes. The analysis revealed a fixed-effects combined effect size (OR* *=* *0.146, 95% CI: 0.084–0.256, *P *= 0.000), indicating that the use of carbon nanoparticle tracers considerably contributed to the identification of parathyroid glands. The preoperative application of carbon nanoparticles resulted in a 14.6% reduction in the rate of parathyroid miscutting compared with that in the control group (Figure 8). These findings demonstrate the effectiveness of carbon nanoparticles in reducing the occurrence of parathyroid miscutting and the subsequent complications.

### 3.7 Number of hypocalcemia cases

**Figure 9:**
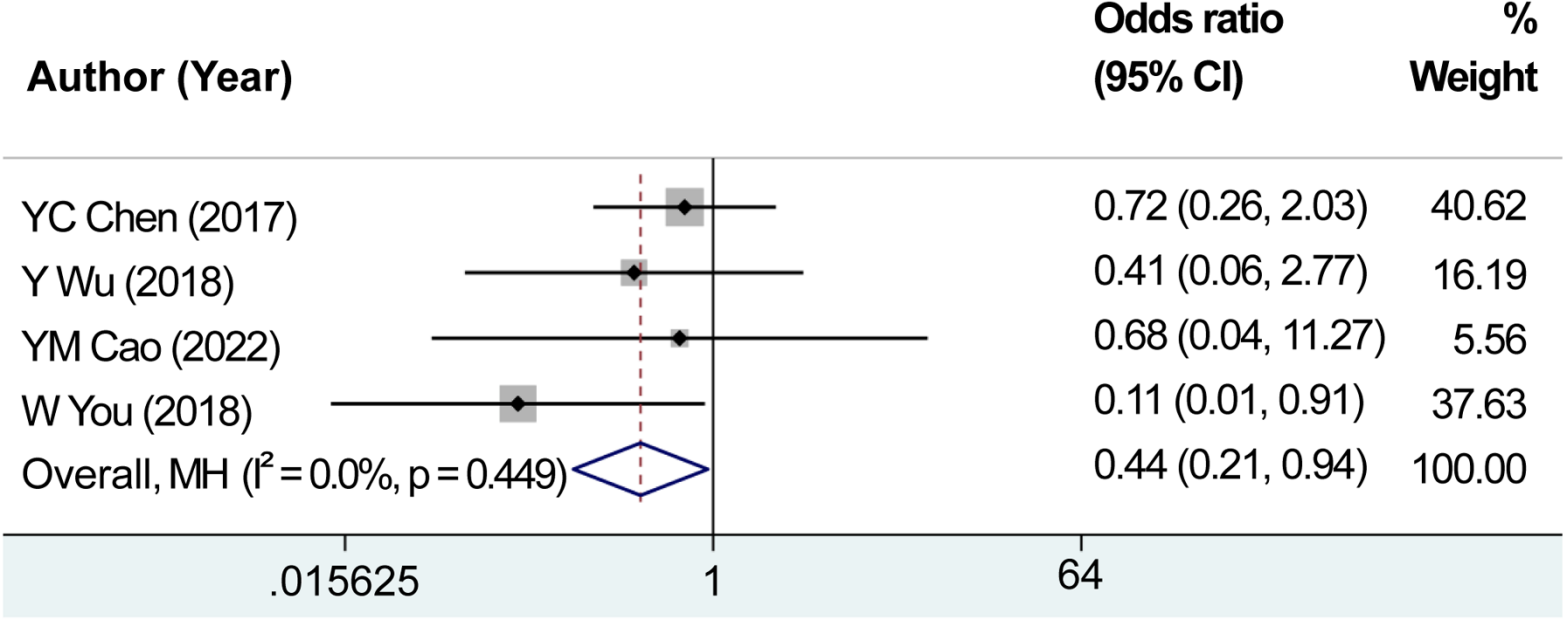
Combined effect size of the number of hypocalcemia cases. The combined effect size using the fixed-effects analysis demonstrated that the utilization of carbon nanoparticle tracers in thyroid surgery reduced the probability of postoperative hypocalcemia by 44% compared with that in the control group.

We analyzed four studies focusing on the number of hypocalcemia cases. With *I*^2^ = 0.00% and *P* = 0.449 for the *Q*-test, the heterogeneity test did not reveal any notable heterogeneity among the studies. Therefore, we chose the fixed-effects methodology for calculating the combined effect sizes. The combined effect size using the fixed-effects analysis (OR* *=* *0.440, 95% CI: 0.206–0.940, *P *= 0.034) demonstrated that the utilization of carbon nanoparticle tracers in thyroid surgery reduced the probability of postoperative hypocalcemia by 44% compared with that in the control group (Figure 9). These findings suggest that the application of carbon nanoparticles in thyroid surgery can effectively prevent postoperative hypocalcemia, reduce its incidence, and improve the safety of thyroid surgery.

### 3.8 Number of recurrent laryngeal nerve injury cases

**Figure 10:**
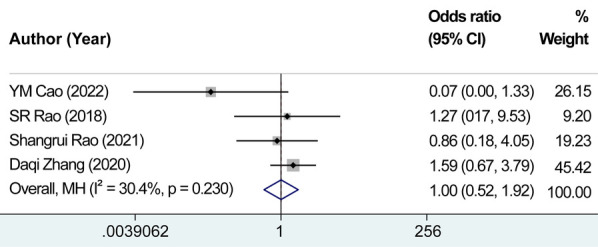
Combined effect size of the number of recurrent laryngeal nerve injury cases. There was no significant difference observed in the mean scores between the experimental and control groups, suggesting that the use of carbon nanoparticle tracers may protect from recurrent laryngeal nerve injuries.

The analysis included four studies focusing on the number of recurrent laryngeal nerve injuries. After testing for heterogeneity, we observed no considerable variation among the studies, with *I*^2^ = 30.4% and *P* = 0.230 for the *Q*-test. Consequently, the fixed-effects model was chosen to estimate the combined effect sizes (OR* *=* *0.440, 95% CI: 0.524–1.925, *P* = 0.990) (Figure 10). Importantly, no significant difference was observed in the mean scores between the experimental and control groups, suggesting that the use of carbon nanoparticle tracers may protect from recurrent laryngeal nerve injuries. However, the preventive effect did not exhibit substantial distinction compared with that of the control group.

## 4 Discussion

This study showed that preoperative applications of nanocarbon in endoscopic thyroid cancer surgery can increase the number of cleared lymph nodes. As a result, it indirectly improves the clearance rate of positive and metastatic lymph nodes, protects the parathyroid glands, reduces the rate of parathyroid miscutting, and lowers the incidence of postoperative hypocalcemia.

In recent years, the global incidence of thyroid carcinoma has considerably increased, making it one of the most widespread endocrine malignancies (1). While most thyroid cancers have a favorable prognosis, studies have revealed that even microscopic thyroid cancers carry a potential risk of lymph node metastasis. Extensive research has consistently demonstrated the significance of cervical lymph node metastasis in evaluating the prognosis of thyroid cancer. Moreover, its occurrence may lead to higher chances of disease recurrence, serve as an indicator for clinical staging, and guide subsequent management strategies. In clinical practice, central and lateral cervical lymph nodes are the most common areas of thyroid cancer lymphatic metastasis (23). Therefore, selecting appropriate surgical methods for thorough lymph dissection plays a crucial role in the treatment effect and prognosis of the disease.

With the continuous progress made in endoscopic surgery technology, endoscopic minimally invasive surgery has also been widely used to treat thyroid cancer. It can ensure the efficacy and safety of surgery and considerably reduce the postoperative scar discomfort and stigma caused by open surgery. Therefore, endoscopic thyroidectomy with ipsilateral lymph node dissection is gradually becoming the mainstream operation for thyroid cancer. Both endoscopic and traditional open surgeries to manage thyroid carcinomas emphasize comprehensive cervical lymphadenectomy, concurrently preserving adjacent structures. Studies have shown that the degree of thorough cervical lymph node dissection directly affects the therapeutic effect of surgery. Therefore, a thorough dissection of the lymph nodes in the thyroid drainage area during surgery is essential. A prerequisite for thorough cervical lymph node dissection would be accurate identification of the lymph nodes. In the past, the distinction between lymph node tissue and non-lymph node tissue usually relied on the surgeon's experience; nonetheless, it might be difficult to provide thorough and accurate assessments just by visual inspection. In addition, the anatomical structure of the tissue around the thyroid varies considerably between individuals, and the parathyroid glands are closely attached to the back of the thyroid gland, making it difficult to accurately distinguish the difference between the two with the naked eye. Hence, the problem of parathyroid miscutting is prone to occur. Unintentional parathyroidectomy during thyroid procedures may result in a range of postoperative complications, including postoperative hypocalcemia, which presents itself through symptoms such as muscle twitches, facial numbness, and cardiac dysfunction, necessitating prolonged pharmacological supplementation, thus markedly affecting individuals' post-surgical quality of life. Studies have found a 5%–25% chance of parathyroid miscutting during thyroid cancer surgery resulting in complications (24). During thyroid cancer surgery, recurrent laryngeal nerve injury could be caused by unclear identification of the recurrent laryngeal nerve, resulting in further complications—postoperative hypocalcemia, hoarseness, and dysphagia.

Carbon nanoparticle technology in thyroid cancer surgery holds immense promise for addressing the previously outlined issues. Currently, the carbon nanoparticles commonly used in clinical practice have the advantages of good targeting, simplicity, and stable properties. Carbon nanoparticles are specially treated smooth spherical particles with 150-nm diameter. After being phagocytosed by macrophages, these nanoparticles can quickly enter the thyroid lymphatic capillaries with a relatively open cell space and accumulate in the lymph nodes in the drainage area, thus staining the lymph nodes and thyroid in the drainage area black. As the gap between endothelial cells of thyroid lymphatic capillaries is smaller than the diameter of carbon nanoparticles, carbon nanoparticle tracers cannot enter the blood, resulting in staining of the parathyroid gland and other peripheral tissues. Applying carbon nanoparticles can help surgeons accurately identify the lymph nodes and thyroid structure in the drainage area in order to increase the number of cleared lymph nodes and avoid postoperative complications caused by the miscutting of the parathyroid gland and recurrent laryngeal nerve injury. Therefore, after approval, carbon nanoparticle tracers have been widely used in clinical thyroid cancer surgery. Adverse effects of carbon nanoparticle injection are relatively rare, and it has a positive effect on the recognition of cervical lymph nodes and protection of parathyroid glands.

In this meta-analysis, we included 13 studies involving 1,463 patients to assess the application effect of carbon nanoparticle tracers in endoscopic thyroid cancer surgery. The analysis focused on six observational indicators, and the discussion is divided into three aspects: the impact of the carbon nanoparticle tracer technique in tracing lymph nodes, protection of parathyroid glands against hypocalcemia, and protective effect on the laryngeal recurrent nerve.

### 4.1 Dissection of traced lymph nodes

According to the meta-analysis, the average quantity of the excised lymph nodes was approximately 3.033 greater within the experimental cohort than the control group following the administration of carbon nanoparticles. As the carbon nanoparticles enter the lymphatic capillary, all lymph nodes in the drainage area of the thyroid gland can be stained. The staining efficiency of carbon nanoparticles was found to be higher than visual inspection alone as it enabled the identification of lymph nodes that were not visually apparent. The analysis revealed that carbon nanoparticle tracers exerted a significant beneficial effect by reducing adverse effects during surgery, thereby facilitating enhanced lymphadenectomy efficiency. However, the ability of carbon nanoparticles to increase the number of lymph nodes retrieved is not always absolute. This is because the clearance of positive lymph nodes can be influenced by the patency of lymphatic capillaries. Additionally, tumor growth can result in a partial blockage of lymphatic capillaries, preventing the desired labeling effect. In the experimental group, 0.457 more positive lymph nodes were cleared and 0.304 more metastatic lymph nodes were identified than those in the control group. However, this effect of carbon nanoparticles on positive lymph nodes and metastatic lymph nodes was not significant probably because the carbon nanoparticles had no significant diagnostic effect on cleared lymph nodes but only marked lymph nodes in the drainage area along the lymphatic capillaries. The use of carbon nanoparticle tracers resulted in a greater number of cleared lymph nodes than traditional methods using visual inspection alone. Additionally, the method led to a slight increase in detecting cancerous and metastasized lymph nodes compared with that in the control group. The present study results corroborate those of Koimtzis et al. (25), suggesting that the utilization of carbon nanoparticles might aid in identifying minute suspect lymph nodes, which will need to be subsequently proven by further studies.

### 4.2 Protecting the parathyroid glands to prevent postoperative hypocalcemia

The results showed that carbon nanoparticles played a major role in the identification of parathyroid glands, and the application of carbon nanoparticle tracers before surgery reduced the parathyroid miscutting rate by 14.6% compared with that in the blank control group. Furthermore, incorporating carbon nanoparticle tracers can markedly decrease the incidence of accidental parathyroidectomy and potentially mitigate the associated postoperative complications. Meanwhile, the probability of postoperative hypocalcemia in the experimental group was reduced by 44% compared with that in blank the group and suggested that the application of carbon nanoparticles in endoscopic thyroid surgery could effectively prevent the occurrence of postoperative hypocalcemia and reduce its incidence, thus improving the safety of surgery. Distinguishing the parathyroid glands from the thyroid tissue is challenging owing to the anatomical structure, which may result in accidental intraoperative parathyroidectomy. The preoperative application of carbon nanoparticles can stain the thyroid gland and lymph nodes in the drainage area. The carbon nanoparticles cannot enter due to the dense interstitial space of the vascular cells. Therefore, without lymphatic capillary drainage, the tissue around the thyroid gland cannot be stained black, resulting in a contrasting black-stained tissue around the gland, thereby reducing the probability of miscutting of the parathyroid glands and the postoperative complications caused by the miscutting of the parathyroid glands. The results of this study were similar to those of Li et al. (26).

### 4.3 Protection of the recurrent laryngeal nerve

In this analysis, the protective effect of carbon nanoparticle tracers on the recurrent laryngeal nerve was not obvious. There was no significant difference in the recurrent laryngeal nerve injury rate between the experimental and control groups. Apart from the included study of Zhang et al. (19), other studies have reported a higher incidence of recurrent laryngeal nerve injury following carbon nanoparticle usage, indicating that these particles offered a degree of neuroprotection. Owing to the limited literature on this issue, more studies on protecting the recurrent laryngeal nerves by carbon nanoparticles should be included for further analysis.

This study has a few limitations. One potential drawback is that it involved a small number of studies, none of which employed blinding methods, affecting how representative the reported findings are. In the future, more randomized, controlled, multi-center, high-quality original studies using blinding methods should be added. Second, the languages of literature searched were only Chinese and English, and there is a lack of original studies reported in other languages and studies on other ethnicities, which might have led to language bias. In addition, the literature included in this study did not focus on the effects of carbon nanoparticles on the recurrence rates and prognosis of thyroid cancer; thus, further research is needed to provide evidence.

## 5 Conclusions

The integration of carbon nanoparticles during endoscopic procedures for thyroid cancer aids in the efficient removal of lymph nodes within the thyroid drainage region. This approach also enhances the identification of pathological lymph nodes and metastases, potentially improving long-term disease outcome and overall patient survival. Application of carbon nanoparticles can also protect the parathyroid glands and reduce postoperative hypocalcemia occurrence and various complications caused by parathyroid miscutting of the parathyroid gland after surgery. These findings suggest that the incorporation of carbon nanotechnology in thyroid surgery holds promise for optimizing therapeutic efficacy and ultimately ensuring more favorable clinical outcomes. However, additional rigorous investigations involving large populations and randomized controlled designs are required in order to confirm these preliminary benefits and fully elucidate the actual impact of carbon nanoparticle technology in this context. The application of carbon nanoparticles can enhance the clearance rate of lymph nodes and protect the tissue around the thyroid.

## Data Availability

The original contributions presented in the study are included in the article/Supplementary Material, further inquiries can be directed to the corresponding author.

## References

[1] Laha D Nilubol N Boufraqech M. New therapies for advanced thyroid cancer. Front Endocrinol. (2020) 11:82. 10.3389/fendo.2020.00082 PMC7257776 32528402

[2] Davies L Welch HG. Increasing incidence of thyroid cancer in the United States, 1973–2002. JAMA. (2006) 295:2164–7. 10.1001/jama.295.18.2164 16684987

[3] Carling T Udelsman R. Thyroid cancer. Annu Rev Med. (2014) 65:125–37. 10.1146/annurev-med-061512-105739 24274180

[4] Carling T Ocal IT Udelsman R. Special variants of differentiated thyroid cancer: does it alter the extent of surgery versus well-differentiated thyroid cancer? World J Surg. (2007) 31:916–23. 10.1007/s00268-006-0837-3 17345120

[5] Guerra A Rosaria Sapio M Marotta V Campanile E Ilaria Moretti M Deandrea M Prevalence of RET/PTC rearrangement in benign and malignant thyroid nodules and its clinical application. Endocr J. (2011) 58:31–8. 10.1507/endocrj.k10e-260 21173509

[6] Lee YS Nam KH Chung WY Chang HS Park CS. Postoperative complications of thyroid cancer in a single center experience. J Korean Med Sci. (2010) 25:541–5. 10.3346/jkms.2010.25.4.541 20357995 PMC2844597

[7] Yang F Jin C Yang D Jiang YJ Li J Di Y Magnetic functionalised carbon nanotubes as drug vehicles for cancer lymph node metastasis treatment. Eur J Cancer. (2011) 47:1873–82. 10.1016/j.ejca.2011.03.018 21493061

[8] Liu X Yu F Wang G Hou L Fan ZY He QQ. Application carbon nanoparticle tracer for lymph node dissection in robotic thyroidectomy. Chin J Gen Surg. (2022) 31:1445–52. 10.7659/j.issn.1005-6947.2022.11.006

[9] Cheng XZ Li YP Qu KP Zhang YP Wang XH. Clinical application of carbon nanoparticle in endoscopic surgery for papillary thyroid carcinoma. J Pract Med. (2020) 36:2235–9. 10.3969/j.issn.1006-5725.2020.16.013

[10] Chen YC Lin XJ Chen HY Chen JB Chen J Lin ZH Application of nano-carbon in lymph node dissection and protection of parathyroid glands in 3D laparoscopic thyroidectomy. China J Endoscopy. (2017) 23:37–41. 10.3969/j.issn.1007-1989.2017.10.008

[11] Wu Y Ding YM Yi SJ. The clinical application of carbon nanoparticles in central compartment lymph node dissection in endoscopic surgery of thyroid carcinoma. Med Sci J Cent South China. (2018) 46:498–500,4. 10.15972/j.cnki.43-1509/r.2018.05.013

[12] Ye K Li XY Chang S Li JD Wang ZM. Clinical application of carbon nanoparticles in endoscopic surgery for thyroid carcinoma. Chin J Gen Surg. (2016) 25:653–8. 10.3978/j.issn.1005-6947.2016.05.005

[13] Yang XH Wang Y Wang P. Application of carbon nanoparticle in endoscopic surgery of thyroid carcinoma. J Laparosc Surg. (2013) 18:262–5. 10.13499/j.cnki.fqjwkzz.2013.04.023

[14] Cao YM Qing JL Wang DY Zhang MM. The application of color ultrasound-guided nano-carbon tracing in minimally invasive surgery of PTC patients. Imaging Sci Photochem. (2022) 40:535–9. 10.7517/issn.1674-0475.211226

[15] You W Jiang B Deng XJ. Application of the nanocarbon suspension tracer technique in laparoscopic radical operation of thyroid cancer. J Baotou Med Coll. (2018) 34:1–3. 10.16833/j.cnki.jbmc.2018.12.001

[16] Rao SR Wang ZL Wang Y Pan ZL Lin Z Yu J. A preliminary study on clinical significance and method of using carbon nanoparticles in endoscopic treatment of papillary thyroid microcarcinoma. Chin J Endocr Surg. (2018) 12:291–3. 10.3760/cma.j.issn.1674-6090.2018.04.007

[17] Ma JJ Zhang DB Zhang WF Wang X. Application of nanocarbon in breast approach endoscopic thyroidectomy thyroid cancer surgery. J Laparoendosc Adv Surg Tech. (2020) 30:547–52. 10.1089/lap.2019.0794 32045316

[18] Rao SR Wang ZL Pan CT Wang Y Lin Z Pan ZL Preliminary study on the clinical significance and methods of using carbon nanoparticles in endoscopic papillary thyroid cancer surgery. Contrast Media Mol Imaging. (2021) 2021:6652315. 10.1155/2021/6652315 33994886 PMC8096569

[19] Zhang DQ Fu YT Dionigi G Hu Y Zhang J Wang T A randomized comparison of carbon nanoparticles in endoscopic lymph node dissection via the bilateral areola approach for papillary thyroid cancer. Surg Laparosc Endosc Percutan Tech. (2020) 30:291–9. 10.1097/sle.0000000000000793 32574006

[20] Wang B Qiu NC Zhang W Shan CX Jiang ZG Liu S The role of carbon nanoparticles in identifying lymph nodes and preserving parathyroid in total endoscopic surgery of thyroid carcinoma. Surg Endosc. (2015) 29:2914–20. 10.1007/s00464-014-4020-x 25761552

[21] Jadad AR Moore RA Carroll D Jenkinson C Reynolds DJM Gavaghan DJ Assessing the quality of reports of randomized clinical trials: is blinding necessary? Control Clin Trials. (1996) 17:1–12. 10.1016/0197-2456(95)00134-4 8721797

[22] Wells G Shea B O'Connell D Peterson J Welch V Losos M The Newcastle–Ottawa Scale (NOS) for Assessing the Quality of Non-randomized Studies in Meta-analysis. (2000). Available at: https://www.ohri.ca/programs/clinical_epidemiology/oxford.asp (Accessed August 16, 2023).

[23] Hughes DT Doherty GM. Central neck dissection for papillary thyroid cancer. Cancer Control. (2011) 18:83–8. 10.1177/107327481101800202 21451450

[24] Youngwirth L Benavidez J Sippel R Chen H. Parathyroid hormone deficiency after total thyroidectomy: incidence and time. J Surg Res. (2010) 163:69–71. 10.1016/j.jss.2010.03.059 20605611

[25] Koimtzis G Stefanopoulos L Alexandrou V Tteralli N Brooker V Alawad AA The role of carbon nanoparticles in lymph node dissection and parathyroid gland preservation during surgery for thyroid cancer: a systematic review and meta-analysis. Cancers (Basel). (2022) 14:4016. 10.3390/cancers14164016 36011009 PMC9407010

[26] Li Y Jian WH Guo ZM Li QL Lin SJ Huang HY. A meta-analysis of carbon nanoparticles for identifying lymph nodes and protecting parathyroid glands during surgery. Otolaryngol Head Neck Surg. (2015) 152:1007–16. 10.1177/0194599815580765 25897006

